# The Function of Inositol Phosphatases in Plant Tolerance to Abiotic Stress

**DOI:** 10.3390/ijms20163999

**Published:** 2019-08-16

**Authors:** Qi Jia, Defeng Kong, Qinghua Li, Song Sun, Junliang Song, Yebao Zhu, Kangjing Liang, Qingming Ke, Wenxiong Lin, Jinwen Huang

**Affiliations:** 1 Key Laboratory for Genetics Breeding and Multiple Utilization of Crops, Ministry of Education/College of Crop Science, Fujian Agriculture and Forestry University, Fuzhou 350002, China; 2 Key Laboratory of Crop Ecology and Molecular Physiology (Fujian Agriculture and Forestry University), Fujian Province University, Fuzhou 350002, China; 3 Putian Institute of Agricultural Sciences, Putian 351144, China; 4 Rice Research Institute, Fujian Academy of Agricultural Sciences, Fuzhou 350018, China

**Keywords:** inositol, phosphatidylinositol, phosphatase, stress, signaling pathway

## Abstract

Inositol signaling is believed to play a crucial role in various aspects of plant growth and adaptation. As an important component in biosynthesis and degradation of *myo*-inositol and its derivatives, inositol phosphatases could hydrolyze the phosphate of the inositol ring, thus affecting inositol signaling. Until now, more than 30 members of inositol phosphatases have been identified in plants, which are classified intofive families, including inositol polyphosphate 5-phosphatases (5PTases), suppressor of actin (SAC) phosphatases, SAL1 phosphatases, inositol monophosphatase (IMP), and phosphatase and tensin homologue deleted on chromosome 10 (PTEN)-related phosphatases. The current knowledge was revised here in relation to their substrates and function in response to abiotic stress. The potential mechanisms were also concluded with the focus on their activities of inositol phosphatases. The general working model might be that inositol phosphatases would degrade the Ins(1,4,5)P_3_ or phosphoinositides, subsequently resulting in altering Ca^2+^ release, abscisic acid (ABA) signaling, vesicle trafficking or other cellular processes.

## 1. Introduction

*Myo*-inositol (Inositol, Ins) and its derivative metabolites are ubiquitous in all eukaryotes as both lipids and soluble compounds playing important roles in stress responses, development, and many other processes [1,2]. Upon environmental stresses, some of them are vital in various signal transduction in plants, especially inositol(1,4,5)triphosphate (Ins(1,4,5)P_3_) and phosphatidylinositol(4,5)bisphosphate (PtdIns(4,5)P_2_) [3,4,5,6,7]. They pass the cellular messages via addition or removal of lipids or phosphates to Ins and its derivatives, which could be mediated by synthases, kinases, phospholipases, and phosphatases [8,9]. Thus, those related enzymes are crucial in the regulation of these signaling pathways. In comparison to the other well-studied enzymes, limited information has been reviewed for the phosphatases in the Ins and phosphatidylinositol (PtdIns) signaling in plants. Here, we focus on these phosphatases and their function in abiotic tolerance.

## 2. The Biosynthesis and Degradation of Inositol and Its Derivatives

**Figure 1:**
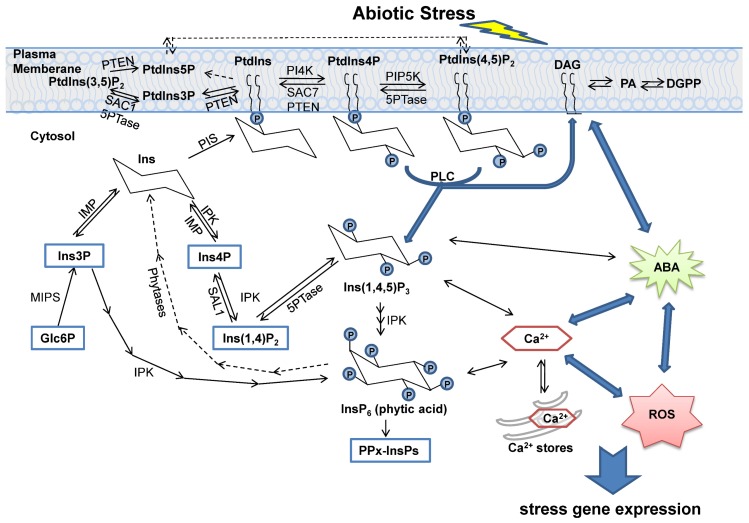
Schematic representation of inositol phosphatases in the plant inositol (Ins) signaling pathways under stress. It illustrated the network of the inositol phosphate (IP) and phosphoinositide (PI) signaling pathway, together with the stress responding processes, such as the ABA pathway, Ca^2+^ release, and ROS generation. The dashed arrows indicated the putative pathways. Ins is soluble, whereas phosphatidylinositol (PtdIns) is bound to the membrane. In the Ins signaling pathways, inositol(1,4,5)trisphosphate (Ins(1,4,5)P_3_, IP_3_), phytic acid (InsP_6_), diacylglycerol (DAG), and phosphatidic acid (PA) are all signaling molecules. ABA—abscisic acid, DGPP—diacylglycerolpyrophosphate, Glc6P—glucose-6-phosphate, IMP—inositol monophosphatase, IPK—inositol polyphosphate multi kinase, MIPS—*myo*-inositol-3-phosphate synthase, P—phosphate, PIP5K—PtIns4P 5-kinase, PI4K—phosphatidylinositol 4-kinase, PIS—phosphatidylinositol synthase, PKC—protein kinase C, PLC—phospholipase C, PPx-InsPs—pyrophosphates, PTEN—phosphatase and tensin homologue deleted on chromosome 10, PtdIns—phosphatidylinositol, ROS—reactive oxygen species, SAC—suppressor of actin, 5PTases—inositol polyphosphate 5-phosphatases.

Inositol could be synthesized from glycolytic glucose-6-phosphate (Glc6P) or be regenerated from various phosphate forms of inositol, which is produced during the metabolism of phosphoinositides. As shown in Figure 1, Glc6P is catalyzed to *myo*-inositol-3-phosphate (Ins3P) by *myo*-inositol-3-phosphate synthase (MIPS). Subsequently, Ins3P is dephosphorylated by inositol monophosphatase (IMP) to form inositol. IMP is also responsible for the dephosphorylation of *myo*-inositol-4-phosphate (Ins4P) [10,11]. Free inositol could be linked to glycerophospholipid to generate the basic inositol containing phospholipid, phosphatidylinositol (PtdIns), by phosphatidylinositol synthase (PIS) [5]. The hydroxyl groups of PtdIns could be phosphorylated at the positions 3, 4, and 5 of the lipid head group sequentially by a series of PtdIns kinases. Unlike the animals, plants have evolved only five phosphorylated isomers, including three PtdIns monophosphates (PtdIns3P, PtdIns4P, PtdIns5P) and two PtdIns bisphosphates (PtdIns(3,5)P_2_, PtdIns(4,5)P_2_). The other two, PtdIns(3,4)P_2_ and PtdIns(3,4,5)P_3_, identified in animals, have not been found in plants [4,12].

On the other hand, PtdIns4P and PtdIns(4,5)P_2_ can be hydrolyzed into diacylglycerol (DAG) and the corresponding phosphoinositide phosphates (PtdInsPs) by phospholipase C (PLC) (Figure 1) [13]. DAG and inositol-1,4,5-trisphosphate (Ins(1,4,5)P_3_, also abbreviated as IP_3_ in this article) are believed as second messages for various signal transduction. In brief, the membrane-localized DAG activates the protein kinase C (PKC) and the soluble InsP_3_ diffuses in cytosol to release Ca^2+^ from intracellular stores via a ligand-gated Ca^2+^ channel [5,14]. DAG can also be used to generate phosphatidic acid (PA), which is also an important signaling molecule [6]. Those inositol polyphosphates can be further phosphorylated by inositol polyphosphate multi kinases (IPKs) and stored as phytic acid (inositol-1,2,3,4,5,6-hexakisphosphate, InsP_6_) in seeds and other storage tissues [4,15]. InsP_6_ has been identified as a signaling molecular to regulate Ca^2+^ release as well [6]. Moreover, InsP_6_ could be converted to pyrophosphates, denoted as PPx-InsPs [16]. Notably under abiotic stress, there are crosstalks between the Ins signaling pathway and phytohormones, especially abscisic acid (ABA) [4,6].

## 3. Phosphatases in Inositol Signaling Pathways

**Figure 2:**
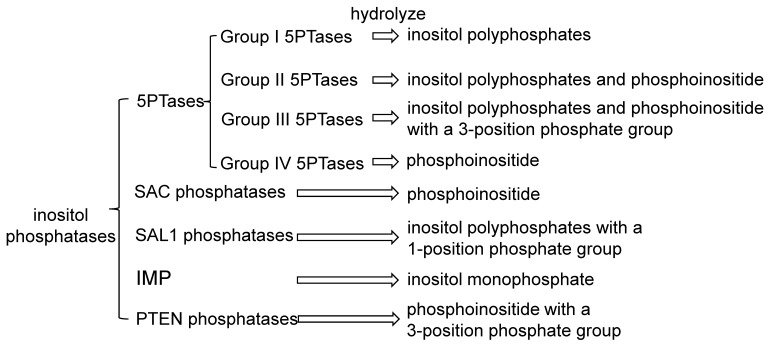
Inositol phosphatases and their inositol-related substrates overviewed in this study. IMP—inositol monophosphatase, PTEN—phosphatase and tensin homologue deleted on chromosome 10, SAC—suppressor of actin, 5PTases—inositol polyphosphate 5-phosphatases.

Among the processes of inositol phosphate (IP) and the phosphoinositide (PI) signaling pathway, dephosphorylation is catalyzed by specific inositol phosphatases on various substrates (Figure 1). Until now, dozens of enzymes have been identified, including inositol polyphosphate 5-phosphatases (5PTases), suppressor of actin (SAC) phosphatases, SAL1 phosphatase/FIERY1 (FRY1) and its homologs, inositol monophosphatase (IMP), and phosphatase and tensin homologue deleted on chromosome 10 (PTEN)-related phosphatases (Figure 2). Most knowledge of them was obtained from the studies in the model plant *Arabidopsis thaliana*. These plant inositol phosphatases have a broad function in development and adaptation by altering the IP or PI signaling pathways. The general information of those *Arabidopsis* proteins was listed in Table 1. Interestingly, one certain inositol phosphatase could hydrolyze several substrates, even both inositol phosphate and phosphoinositide. One substrate could be degraded by more than one enzyme as well, suggesting their redundant roles in multiple aspects of life processes.

The 5PTases family is the biggest family of the mentioned inositol phosphatases, containing 15 members in *Arabidopsis*, 21 in rice, and 39 in soybean [17]. 5PTases hydrolyze the phosphate bond on the 5-position of the inositol ring from both inositol phosphate and phosphoinositide with the conserved inositol polyphosphate phosphatase catalytic (IPPc) domain. Due to the substrate specificity, mammalian 5PTases have been classified into four groups [18]. Group I, 5PTases hydrolyze only the water-soluble inositol polyphosphates (Ins(1,4,5)P_3_ and Ins(1,3,4,5)P_4_); group II the water-soluble inositol polyphosphates and the membrane-bound phosphoinositide; group III, Ins(1,3,4,5)P_4_ and PtdIns(3,4,5)P_3_ with a 3-position phosphate group; and group IV only phosphoinositide. Similar as the mammalian counterparts, plant 5PTases also have various substrate specificities. The substrates have been identified by biochemical evidences for twelve of the fifteen *Arabidopsis* 5PTases, including Group I, Group II, and Group IV 5PTases (Table 1). Since several 5PTases could hydrolyze Ins(1,4,5)P_3_ to prevent its accumulation, it is believed to terminate the corresponding Ins(1,4,5)P_3_ pathway and alter abscisic acid (ABA) signaling, Ca^2+^ release, and reactive oxygen species (ROS) production [19,20,21].

The SAC phosphatases are polyphosphoinositide phosphatases, containing the enzymatic SAC domain [22]. There are nine members in *Arabidopsis* [23]. Most *Arabidopsis* SAC phosphatases have a ubiquitous expression pattern, except for AtSAC6 which is only expressed in flowers under normal growth condition. Their expression was not altered by treatment with phytohormones (auxin, cytokinin, GA, and ABA) [23]. When two-week-old seedlings were treated with various stresses (dark, cold, salt, and wounding), only *AtSAC6* has been identified to be induced by salt stress, indicating it would be involved in salt response [23]. Besides, the *sac9* mutants exhibit a constitutive stress response with highly up-regulated stress-induced genes and over-accumulation of ROS [24]. Though there is limited knowledge on their substrate specificity, SAC phosphatases have been found to affect the accumulation of some certain phosphatidylinositol phosphates, such as PtdIns(4,5)P_2_, PtdIns(3,5)P_2_, and PtdIns4P, in addition to having a possible role in vesicle trafficking [24,25,26].

Comparing to 5PTases and SAC phosphatases, there are fewer members in the SAL, IMP, and PTEN families and most of them behave as bifunctional enzymes (Table 1). AtSAL1 and AtSAL2 exhibit the activities of not only inositol polyphosphate 1-phosphatase but also 3’(2’),5’-bisphosphate nucleotidase [47,56]. The other SAL1 homologues without inositol phosphatases are not listed here. AtSAL1 has been identified as an important player in response to various stresses, probably through both enzyme activities [48,49,51,53,63,64,65]. Three IMP members have been identified in *Arabidopsis* [11]. IMP and inositol monophosphatase-like 1 (IMPL1) exhibit bifunctional activities affecting both inositol and ascorbate synthesis pathways, whereas IMPL2 is a histidinol-phosphate phosphatase affecting histone biosynthesis pathways [57,66]. The IMPs from other plants have been shown to play a role in stress tolerance [67,68,69], which we will discuss later. PTEN members are also dual phosphatases for protein and phosphoinositide phosphates [62]. The transcript and protein analyses showed that *AtPTEN2a* and *AtPTEN2b* were up-regulated at transcriptional level, but not at protein level under salt and osmotic stress [62], suggesting their potential roles in plant adaptation to stress. But no further evidence has been reported yet.

## 4. Function of Inositol Phosphatases under Abiotic Stress

### 4.1. 5PTases and Plant Responses to Abiotic Stress

The capacity of 5PTases hydrolyzing IP_3_ is believed to be vital in the termination of IP_3_, consequently altering Ca^2+^ oscillations, ABA signaling, and other stress-related pathways. The transgenic *Arabidopsis* plants overexpressing mammalian type I (group I) inositol polyphosphate 5-phosphatase (InsP 5-ptase) exhibited increased drought tolerance with less water loss [70]. The contents of IP_3_ and IP_6_ were decreased in the transgenic lines as expected, thus attenuating ABA induction and Ca^2+^ signal transduction. The stomata were less responsive to the inhibition of opening by ABA and more sensitive to ABA-induced closure. Furthermore, the microarray data showed that *dehydration-responsive element-binding protein 2A* (DREB2A), encoding a drought-inducible ABA-independent transcription factor, and the DREB2A-regulated genes were induced in the InsP 5-ptase transgenic plants, suggesting the drought tolerance is mediated via the DREB2A-dependent pathway [70].

For plant 5PTases, it is common to take a role in the degradation process of inositol phosphate or phosphoinositide, terminating the IP_3_ signaling, thus altering of ABA pathway and Ca^2+^ release, which is believed to be vital in stress tolerance [19,21,34,39]. However, only three of the 15 At5PTases have been identified to play important roles in abiotic stress with genetic and biochemical evidences until now. At5PTase7 and At5PTase9 function in salt tolerance, and At5PTase13 in low nutrient and sugar stress [35,36,41].

The T-DNA insertion mutants of *At5PTase7* or *At5PTase9* increased salt sensitivity and the overexpression plants increased salt tolerance [35,36]. Mutation in either *At5PTase7* or *At5PTase9* reduced ROS production in the *Arabidopsis* roots after 10 to 15 min after salt treatment. Additionally, the expression of salt-responsive genes, such as *RD29A* and *RD22*, was not induced highly in both mutants as in the wild-type under salt stress [35,36]. It suggested that the defect in *At5PTase7* or *At5PTase9* would disturb ROS production and salt-responsive gene expression, probably hampering the subsequent rescue signal transduction. Interestingly, the *At5PTase9* mutants appeared to have a better ability to resistant osmotic stress. Meanwhile, the *At5PTase9* mutants decreased Ca^2+^ influx and fluid-phase endocytosis [36]. Though the At5PTase7 and At5PTase9 isomers take non-redundant roles in regulating plant responses to salt stress, they share the same substrates, membrane-bound phosphoinositide, indicating that phosphoinositide would be important in salt tolerance [36].

At5TPase13 is one of the four At5TPases (At5TPase12-15), which contain the plant specific WD40 repeats [38,44]. The T-DNA insertion mutants of *At5TPase13* showed a reduction of root growth under limited nutrient conditions and germination rates in response to sugar stress, along with ABA insensitivity [41]. The yeast two-hybrid analyses suggested that its WD40 repeat domain interacts with the sucrose nonfermenting-1-related kinase (SnRK1.1), which is an energy/stress sensor [41]. The genetic and biochemical evidences indicated that At5TPase13 acts as a positive regulator of SnRK1.1 under low-nutrient or low-sugar conditions, as a negative regulator under severe starvation conditions through affecting the proteasomal degradation of SnRK1.1. Strangely, the *At5ptase13* mutants accumulate less IP_3_ in response to sugar stress [41]. Again, At5PTase13 could alter cytosolic Ca^2+^ to regulate PHOYOTROPIN1 signaling under blue light [40].

Besides, several transcriptional analyses showed that the expression of multiple At5PTases is greatly up- or down-regulated in response to a series of abiotic stresses, such as cold, osmotic, salt, drought, oxidative, and heat [35,36,71]. Considering the general function of the known 5PTases in the inositol pathway, Ca^2+^ signaling, ABA responses, ROS generation, vesicle trafficking, and possible connection with other phytohormones [43,71], it could imply their potential roles in plant responses to abiotic stress.

### 4.2. SAL1 and Plant Responses to Abiotic Stress

AtSAL1, identified as a homologue of the yeast HAL2 in *Arabidopsis* and also well-known as FIERY1 (FRY1), has dual enzymatic activity of inositol phosphatase and nucleotidase, which play a role in both inositol signaling and nucleotide metabolite [47,48]. AtSAL1 functions broadly in responses to abiotic stresses, including salt, cold, lithium, drought, cadmium, high light, and oxidative, probably with the contributions of both enzymatic activity [48,49,51,52,53,63,72,73]. Here we will focus on its activity of inositol polyphosphate 1-phosphatase. Remarkably, it can hydrolyze the signaling molecular IP_3_, thus affecting the subsequent steps in a similar pattern of 5PTases, which we have discussed above.

It seems the effects of AtSAL1 on stress responses are controversial. Ectopic expression of *AtSAL1* could increase lithium tolerance in yeast by modifying Na^+^ and Li^+^ effluxes [47]. Ectopic expression of its homologue in soybean, *GmSAL1*, could alleviate salinity stress in tobacco BY-2 cells [74]. Mutation in *AtSAL1* would cause more sensitivity to salt, osmotic, and cold stress in *Arabidopsis* [48,72]. However, another *Atsal1* mutant, *hos2* with a single amino acid substitution exhibited as more resistant to lithium and salt stress [72]. Moreover, overexpression of *AtSAL1* or ectopic expression of *GmSAL1* could not enhance salt tolerance in *Arabidopsis* [49,74]. Loss function in *AtSAL1* would enhance drought and cadmium resistance in *Arabidopsis*, suggesting it would be a negative regulator of stress tolerance [51,63]. Expressing the modified *SAL1,* by inserting the META motif from black yeast *Aureobasidium pullulans*, *ApHal2*, improved salt and drought tolerance in *Arabidopsis* [73]. It seems the presence of the META motif should be responsible for its ability on the stress tolerance, but the mechanism is still obscure.

The molecular mechanism of AtSAL1 in stress responses seems to be complicated for its multiple effects in various cellular processes. First, AtSAL1 would regulate stress tolerance and ABA responses via IP_3_ signaling. The *Atsal1* mutants increase IP_3_ accumulation and the expression levels of ABA and stress genes, including *RD29A*, cold-specific *CRT-binding factor 2* (*CBF2*), and *CBF3* [48]. On the contrary, ectopic expression of *GmSAL1* leads to a reduction of IP_3_ accumulation and suppression of the ABA-induced stomatal closure [74]. Furthermore, it also showed AtSAL1 could regulate Ca^2+^ release and modulate the auxin pathway by IP_3_ signaling in plant development [54,55]. It seems a similar consequence of AtSAL1 in the IP_3_ signaling as for 5PTases. Maybe further investigation will supply evidence that AtSAL1 takes a role in Ca^2+^ release and its downstream signaling in response to abiotic stress as well. Secondly, AtSAL1 also regulates the ion homeostasis via the IP_3_ pathway. Ectopic expression of *AtSAL1* could modify Na^+^ and Li^+^ effluxes in yeast for lithium and salt tolerance [47]. *GmSAL1*-transgenic BY-2 cells could compartmentalize more Na^+^ in vacuolar for protection from salt stress [74]. Additionally, AtSAL1 takes a role more likely as a phosphoadenosine phosphatase under drought, high light, and oxidative stress, for only 3′-phosphoadenosine 5′-phosphate (PAP), not IP_3_, accumulated in the *Atsal1* mutants, when suffering stresses [49,52,53,65]. The genetic evidences indicated that PAP accumulation could also affect the ABA pathway, relying on, rather, the negative regulator ABH1 in the branched ABA pathway, than ABI1 in the core ABA pathway [49]. AtSAL1 could protect 5′ to 3′ exoribonucleases (XRNs) by degrading PAP and subsequently modulate the expression of the corresponding nuclear genes, supposed as the chloroplast retrograde pathway [52,53,65]. Besides, the *AtSAL1*-deficient mutants have been found to attenuate endoplasmic reticulum (ER) stress under cadmium stress [63]. But no exploration has been made to determine its connection with the IP3 signaling or SAL1-PAP pathway. This would provide a new insight on the mechanism of AtSAL1 in various stress tolerance [63].

### 4.3. IMPs and Plant Responses to Abiotic Stress

IMPs were first identified in tomato to play a role in inositol synthesis with high sensitivity to lithium [10]. Their homologues in *Arabidopsis* have also been characterized as multi-functional enzymes involved in inositol, ascorbate, and histone biosynthesis [57,59,66], so do their homologues in other plants, such as rice (*Oryza sativa L.*), chickpea (*Cicer arietinum L.*), soybean (*Glycine max*), barley (*Hordeum vulgare*), and *Medicago truncatula* [68,69,75]. The genetic studies showed that IMPs play a role in seed development in *Arabidopsis* [11]. Chickpea IMP could also influence seed size/weight [76]. But few explorations have been made with *Arabidopsis* IMP on stress tolerance yet. Only some authors have tried assays in chickpea and rice suggesting that IMPs also function in response to abiotic stress [67,68,69]. But it is still unclear how IMPs influence the inositol pathway to confer stress.

Biochemical evidence demonstrated that CaIMP contains the same enzyme activity as *Arabidopsis* IMP and IMP activity is increased in chickpea seedlings under abiotic stresses, including salt, cold, heat, dehydration, and paraquat. It is consistent with the results of the transcript analyses by qRT-PCR, which showed that *CaIMP* is induced under abiotic stress and ABA treatment [69]. The *CaIMP*-transgenic *Arabidopsis* plants exhibited enhanced tolerance to abiotic stress, whereas the *IMP*-deficient *Arabidopsis* mutants increased the sensitivity to stress during seed germination and seedling growth. The inositol content and ascorbate content of the *CaIMP*-overexpressing lines are higher than the wild-type and the vector control, suggesting CaIMP would improve the plant tolerance to stress through both metabolic pathways [69]. Association analyses performed with 60 chickpea germplasm accessions showed that NCPGR90, a simple sequence repeat marker for phytic acid content and drought tolerance, is located to the 5’UTR of *CaIMP* [68]. The transcript lengths of *CaIMP* are different between the drought-tolerant and drought-susceptible accessions, suggesting this variation might regulate phytic acid contents in plants, thus conferring drought tolerance in chickpea [68]. In another study, this variation also causes the differential protein level and enzymatic activity of CaIMP [76].

Rice *OsIMP* is significantly upregulated by cold and ABA treatment by transcript analyses [67]. The promoter analyses on sequence also identified several important stress-responding cis-acting elements, including ABRE-element (abscisic acid responsiveness), LTR (low-temperature responsiveness), TCA-element (salicylic acid responsiveness), GARE-motif (gibberellins responsive), and MBS (MYB binding site). Ectopic expression of *OsIMP* in tobacco improved cold tolerance. The transgenic plants contained more inositol content, less hydrogen peroxide (H_2_O_2_), and less malondialdehyde (MDA), with increased antioxidant enzyme activities under normal and cold stress conditions [67]. It suggested that the accumulation of inositol by expressing *OsIMP* would modulate the antioxidant enzymes’ activities to conquer cold stress.

## 5. Conclusions

Substantial evidences demonstrate inositol phosphates, phosphoinositides, and the related inositol signaling play a crucial role in various life processes of development and environmental adaptation in plants [1,4,6,7,12]. When plants suffer abiotic stress from the environment, a membrane receptor would accept the stimulus and the membrane-associated phosphoinositides would pass the cellular message by producing second messages, lipid-bound DAG, and soluble IP_3_. Components involved in the inositol pathways have been noted for their general roles in stress tolerance. This article focused on the knowledge about inositol phosphatases, which are considered to be more important in the degradation pathway of IP_3_ signaling, and their function in plant responses to abiotic stress.

Around 30 members of inositol phosphatases from five families have been identified. Their functions and mechanisms are still largely unknown. Biochemical and physiological data, especially those from analytical techniques, have delineated their substrates and the affecting signals. Moreover, the genetic evidences elucidate the genes’ function and how to pass the signals. In general, loss-in-function of inositol phosphatases usually cause the accumulation of IP_3_ or phosphoinositides, thus facilitating Ca^2+^ release from cellular stores and affecting ABA or other phytohormones’ pathways. For their effects on lipid-bound phosphoinositides, several enzymes have been proved to be involved in vesicle trafficking. For most of the inositol phosphatases, the existed evidences could only support part of the model. There are also some other puzzles. Since phytic acid (InsP_6_) could also serve as a signaling molecule to regulate Ca^2+^ release [6], what is the role of inositol phosphatases in this process? There are multiple genes in the same family, especially 5PTases and SAC phosphatases. How do plants coordinate their function? Most of the knowledge about these enzymes is obtained from the mode plant *Arabidopsis*. Study from other plants is relatively rare. Do these inositol phosphatases take a universal role in all plants under abiotic stress? Hopefully, more exploration will expand our understanding about inositol phosphatases.

## Figures and Tables

**Table 1:** Phosphatases of the inositol signaling pathway in *Arabidopsis thaliana*.

Name	Gene ID	Substrates	Cellular Localization	Expression Patterns	Function	References
5PTase—hydrolyze inositol-5-phosphate						
At5TPase1	At1G34120	Group II Ins(1,4,5)P_3_, Ins(1,3,4,5)P_4_, PtdIns(4,5)P_2_	-	leaf, flower, bolt, seedling	alter ABA and light signaling, stomatal opening, seedling development	[19,21,27,28]
At5TPase2	At4G18010	Group II Ins(1,4,5)P_3_, Ins(1,3,4,5)P_4_, PtdIns(4,5)P_2_	-	leaf, flower, bolt, seedling	alter ABA signaling, seedling development	[20,27,28]
At5TPase3	At1G71710	Group II PtdIns(4,5)P_2_, PtdIns(3,4,5)P_3_, Ins(1,4,5)P_3_, Ins(1,3,4,5)P_4_,	-	-	-	[29]
At5TPase4	At3G63240	Group IV PtdIns(4,5)P_2_	-	-	-	[29]
At5TPase5/MRH3/BST1	At5G65090	-	-	-	root hair development	[30,31]
At5TPase6/CVP2	At1G05470	Group IV PtdIns(4,5)P_2_, PtdIns(3,4,5)P_3_	-	vascular system	foliar vein patterning, root branching	[32,33,34]
At5TPase7/ CVL1	At2G32010	Group IV PtdIns(4,5)P_2_, PtdIns(3,4,5)P_3_	plasma membrane, nuclear speckles	vascular system	foliar vein patterning, root branching, salt tolerance, and ROS production	[33,34,35]
At5TPase8	At2G37440	-	-	-	-	-
At5TPase9	At2G01900	Group IV PtdIns(4,5)P_2_, PtdIns(3,4,5)P_3_	-	root	salt tolerance and ROS production endocytosis	[36]
At5TPase10	At5G04980	-	-	-	-	-
At5TPase11	At1G47510	Group IV PtdIns(4,5)P_2_, PtdIns(3,5)P_2_, PtdIns(3,4,5)P_3_	cell surface or plasma membrane	flower, leaf, root, silique, bolt, seedling	seedling development	[27,37]
At5TPase12	At2G43900	Group I Ins(1,4,5)P_3_	-	pollen grain, leaf and flower (mostly); root, stem and young seedling (weakly)	pollen dormancy/germination	[38,39]
At5TPase13	At1G05630	Group I Ins(1,4,5)P_3_,	nucleus	young seedlings, flowers	cotyledon vein development, alter auxin, ABA, sugar and PHOTOTROPIN1 signaling, root gravitropism, vesicle trafficking	[38,39,40,41,42,43]
At5TPase14	At2G31830	Group II PtdIns(4,5)P_2_, PtdIns(3,4,5)P_3,_ Ins(1,4,5)P_3_	-	pollen grain	-	[38,39]
At5TPase15/ FRA3	At1G65580	Group II PtdIns(4,5)P_2_, PtdIns(3,4,5)P_3_, Ins(1,4,5)P_3_	-	seedling, stem, root, flower, mature leaf (weak)	secondary wall synthesis and actin organization	[44]
SAC—hydrolyze phosphatidylinositol phosphates						
SAC1/FRA7	At1G22620	PtdIns(3,5)P_2_	Golgi	ubiquitous, predominant in vascular tissues and fibers of stems	cell morphogenesis, cell wall synthesis, actin organization	[23,45]
SAC2	At3G14205	-	tonoplast	ubiquitous	vacuolar function	[23,26]
SAC3	At3G43220	-	tonoplast	ubiquitous	vacuolar function	[23,26]
SAC4	At5G20840	-	tonoplast	ubiquitous	vacuolar function	[23,26]
SAC5	At1G17340	-	tonoplast	ubiquitous	vacuolar function	[23,26]
SAC6/SAC1b	At5G66020	-	endoplasmic reticulum	pollen grain	embryo development	[22,23]
SAC7/SAC1c/RHD4	At3G51460	PtdIns4P	endoplasmic reticulum	most tissues (strong)	embryo development, root hair development	[22,23,25]
SAC8/ AtSAC1a	At3G51830	-	endoplasmic reticulum	hypocotyls of seedlings, pollen grain, most tissues (week),	embryo development	[22,23]
SAC9	At3G59770	-	-	root (strong), leaf and shoot (weak)	cell wall formation, stress response	[23,24,46]
SAL—hydrolyze inositol-1-phosphate						
AtSAL1/ AtFIERY1 (AtFRY1)/ HOS2/RON1	At5G63980	Ins(1,4)P2, Ins(1,3,4)P3, PAP, PAPS	chloroplast, mitochondria	vascular tissue	alter ABA, auxin and stress signaling (cold, drought, salt, lithium, high light, cadmium), venation patterning	[47,48,49,50,51,52,53,54,55]
AtSAL2	At5G64000	Ins(1,4)P_2_, PAP,	-	-	-	[56]
IMP—hydrolyze inositol-3-phosphate, inositol-4-phosphate						
IMP/VTC4	At3G02870	Ins3P, Ins1P, L-Galactose-1-P	cytosol	photosynthetictissues	seed development, ascorbate biosynthesis, alter cold, salt and ABA responses	[11,57,58,59]
IMPL1	At1G31190	Ins3P, Ins1P, Ins2P, L-Galactose-1-P	chloroplast	ubiquitous	seed development	[11,57,59]
IMPL2	At4G39120	Histidinol 1-P	chloroplast	root (strong), hypocotyl (weak)	seed development, histidinebiosynthesis	[11,57,59]
PTEN—hydrolyze inositol-3-phosphate						
PTEN1	At5G39400	PtdIns(3,4,5)P_3_, phosphotyrosin	vesicles, autophagic body	pollen grain	pollen development	[60,61]
PTEN2a	At3G19420	PtdIns3P, PtdIns(3,4)P_2_, PtdIns(3,5)P_2_, PtdIns4P, PtdIns(3,4,5)P_3_, phosphotyrosin	-	seedling, leaf, flower, silique	-	[62]
PTEN2b	At3G50110	PtdIns3P, phosphotyrosin	-	seedling, leaf, flower, silique	-	[62]

BST1, BRISTLED1; CVL1, CVP2-like1; CVP2, cotyledon vascular pattern2; FRA3, fragile fiber 3; FRA7, fragile fiber 7; HOS2, high expression of osmotic stress-regulated gene expression 2; IMP, *myo*-inositol monophosphatase; Ins, inositol, MRH3, root hair morphogenesis 3; P, phosphate; PAP, 3’-phosphoadenosine 5′-phosphate; PAPS, 2’-PAP and 3’-phosphoadenosine 5’-phosphosulfate; PTEN, phosphatase and tensin homologue deleted on chromosome 10, PtdIns, phosphatidylinositol, RHD4, root hair defective 4; RON1, rotunda 1; SAC, suppressor of actin, VTC4, vitamin C 4, 5PTases, inositol polyphosphate 5-phosphatases.

## Abbreviations

ABA	abscisic acid
ABRE	abscisic acid responsiveness
BST1	BRISTLED1
CBF	CRT-binding factor
CVL1	CVP2-like1
CVP2	cotyledon vascular pattern2
DAG	diacylglycerol
DGPP	diacylglycerol pyrophosphate
DREB2A	dehydration-responsive element-binding protein 2A
ER	endoplasmic reticulum
FRA3	fragile fiber 3
FRA7	fragile fiber 7
FRY1	FIERY1
Glc6P	glucose-6-phosphate
GARE	gibberellins responsive
HOS2	high expression of osmotic stress-regulated gene expression 2
IMP	*myo*-inositol monophosphatase
IMPL	inositol monophosphatase-like
Ins	inositol
InsP 5-ptase	inositol polyphosphate 5-phosphatase
IP	inositol phosphate
IP_3_	Inositol(1,4,5)trisphosphate
IPK	inositol polyphosphate multi kinase
IPPc	inositol polyphosphate phosphatase catalytic
LTR	low-temperature responsiveness
MBS	MYB binding site
MDA	malondialdehyde
MRH3	root hair morphogenesis 3
MIPS	*myo*-inositol-3-phosphate synthase
P	phosphate
PA	phosphatidic acid
PAP	3′-phosphoadenosine 5’-phosphate
PAPS	2’-PAP and 3’-phosphoadenosine 5’-phosphosulfate
PI	phosphoinositide
PIP5K	PtIns4P 5-kinase
PIP4K	PtIns4P 4-kinase
PIS	phosphatidylinositol synthase
PKC	protein kinase C
PLC	phospholipase C
PPx-InsPs	pyrophosphates
PTEN	phosphatase and tensin homologue deleted on chromosome 10
PtdIns	phosphatidylinositol
RHD4	root hair defective 4
RON1	rotunda 1
ROS	reactive oxygen species
SAC	suppressor of actin
SnRK1.1	sucrose nonfermenting-1-related kinase
TCA	salicylic acid responsiveness
VTC4	vitamin C 4
XRN	5′ to 3′ exoribonuclease
5PTases	inositol polyphosphate 5-phosphatases
